# Supplementary material for: Molecular mechanism of influenza A NS1-mediated TRIM25 recognition and inhibition
Source: Nat Commun. 2018 May 8;9:1820. doi: 10.1038/s41467-018-04214-8 (PMC5940772; doi:10.1038/s41467-018-04214-8)
Supplement: Supplementary file 1 — Supplementary Information [file 41467_2018_4214_MOESM1_ESM.pdf]

Supplementary Information for

Molecular mechanism of Influenza A NS1-mediated TRIM25 recognition and inhibition

Koliopoulos *et al.*

This PDF includes:

Supplementary Tables 1 and 2

Supplementary Figures 1-9

Supplementary References

	Human TRIM25 PRYSPRY (434-630) Native PDB: 6FLM	PRYSPRY Mercury derivative
**Data collection**		
Space group	*P*321	*P*321
Cell dimensions		
a, *b*, *c* (Å)	152.75, 152.75, 68.96	152.24, 152.24, 68.81
α, β, γ (°)	90.0, 90.0, 120.0	90.0, 90.0, 120.0
Resolution (Å)	50.0-2.01 (2.10-2.01)*	50.0-2.50 (2.59-2.50)*
R_{sym}	0.124 (0.336)*	0.098 (0.337)*
$I/\sigma I$	11.42 (2.50)*	12.14 (4.83)*
Completeness (%)	84.1 (51.5)* (99.5 at 2.35Å)	98.6 (98.8) *
Redundancy	5.34 (2.36)*	3.83 (3.86)*
**Refinement**		
Resolution (Å)	50.0-2.01	
No. reflections	49981	
$R_{work/}$ R_{free}	0.165/0.206	
No. atoms		
Protein	4845	
Water	511	
Ions	66 (13 x SO_4, 1 Cl)	
B-factors		
Mean	27.5	
R.m.s deviations		
Bond lengths (Å)	0.005	
Bond angles (º)	0.707	

*Values in parentheses are for highest-resolution shell.

Supplementary Table 1: Crystallographic data collection and refinement statistics for the human TRIM25 PRYSPRY domain.

**a) Sample details**	TRIM25 CC-PRYSPRY
Organism	*Homo sapiens*
Source	*Trichoplusia ni*
UniProt sequence ID (residues in construct)	Q14258 (189-630)
Extinction coefficient (A_{280}, 0.1 % (w/v)	0.998
Partial specific volume from chemical composition (cm^3 g^{-1})	0.737
Particle contrast from sequence and solvent constituents, $\Delta\rho$ ($\rho_{protein}$ - $\rho_{solvent}$; 10^{10} cm^{-2})	2.69 (12.20-9.41)
M from chemical composition (Da)	49952 (monomer), 99904 (dimer)
Average *C* in merged data (mg ml^{-1})	5.8 (1.0 – 20)
Solvent	20 mM Na_2HPO_4, 150 mM NaCl, pH 6.5
**b) SAXS data collection parameters**	
Instrument	ESRF BM29 BioSAXS
Wavelength (Å)	0.992
Beam size (µm)	700 x 700
Detector distance (m)	2.867
q measurement range ($Å^{-1}$)	0.0034-0.494
Absolute scaling method	Comparison with scattering from 1 mm pure H_2O
Monitoring for radiation damage	Data frame-by-frame comparison
Exposure time	1 s per frame
Sample temperature (°C)	20
**c) Software employed for SAXS data reduction, analysis and interpretation**	
SAXS data reduction	*I(q)* versus *q* using BsxCuBE, solvent subtraction, averaging and merging using PRIMUS (ATSAS 2.8.2.
Extinction coefficient estimate	ProtParam
Calculation of $\Delta\rho$ and *v* values	MULCh 1.1
Basic analyses: Guinier, *P(r)*, V_P	PRIMUS (ATSAS 2.8.2)
Atomic structure modelling	CNS<br>CRYSOL (ATSAS 2.8.0)
Three-dimensional graphic model representation	PyMOL1.7
**d) Structural parameters**	
Guinier analysis	
I(0)	142.75 ± 0.36
R_g (Å)	69.8 ± 0.04
q_{min} ($Å^{-1}$)	0.0058
qR_g max (q_{min} = 0.0058 $Å^{-1}$)	1.28
Coefficient of correlation, R^2	0.99
M from *I*(0) (ratio to predicted)*	142750 (1.43)
P(r) analysis	
I(0)	142.65± 0.13
R_g (Å)	70.9 ± 0.02
d_{max} (Å)	253
q range ($Å^{-1}$)	0.0058-0.122
X^2 (total estimate from GNOM)	0.52

M from *I*(0) (ratio to predicted)*	142650 (1.43)
Porod volume ($Å^{-3}$) (ratio V_P/calculated *M*)	222000 (2.2)

*The discrepancy between theoretical molecular weight and calculated from I(0) is a result of concentration measurement uncertainties

Supplementary Table 2: Small-angle X-ray scattering statistics according to Trewhella et al.[1]

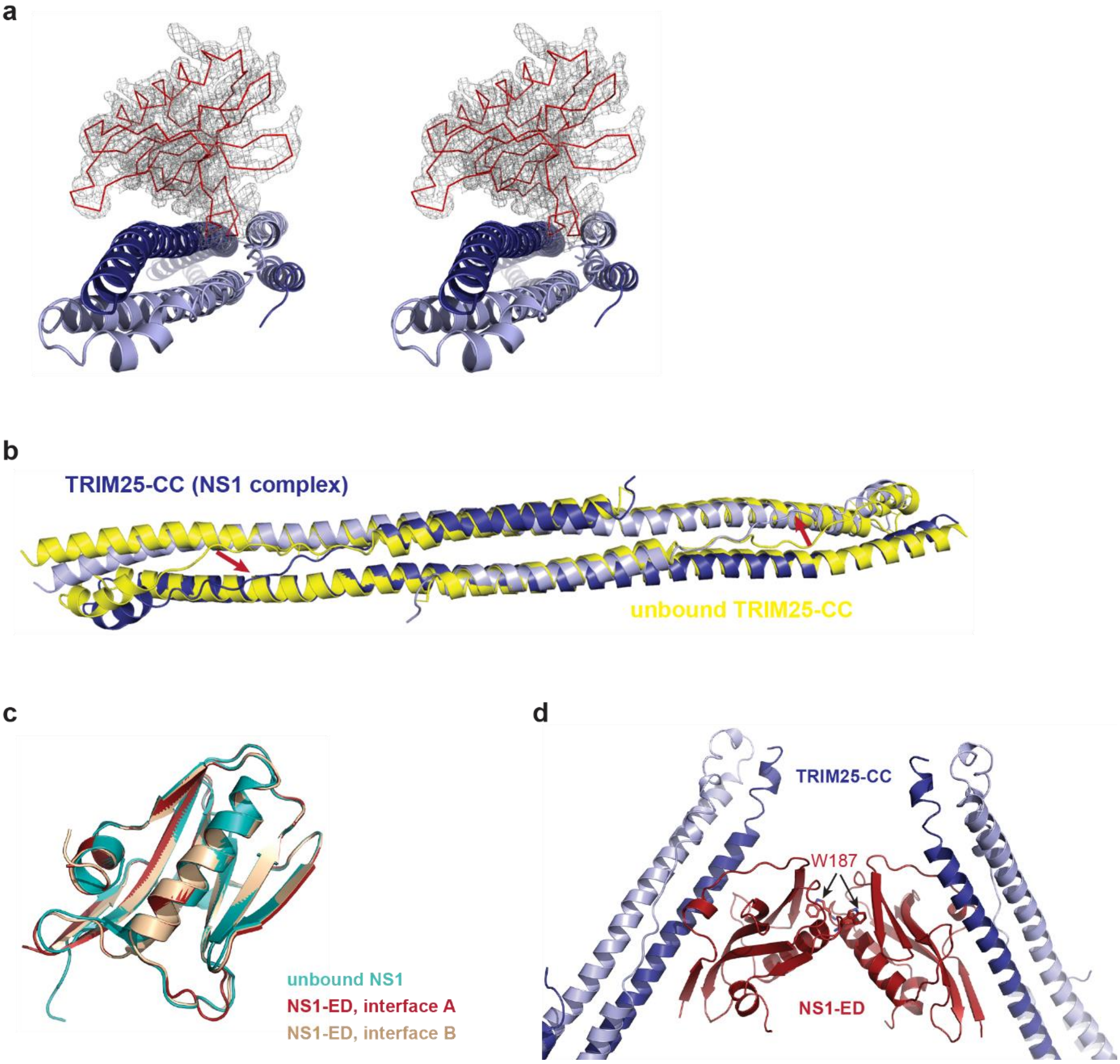

Supplementary Figure 1. Structural alignments of bound and unbound TRIM25-CC and NS1-ED and the NS1-ED W187 interface.

a. 2Fo-Fc electron density map contoured at 2.0 sigma for the NS1 ED domain with the TRIM25-CC in cartoon representation, shown as a cross-eye stereo image. **b.** Structural alignment of TRIM25-CC from the NS1-ED-bound structure (in blue/light blue) and unbound TRIM25-CC (PDB: 4LTB, in yellow). **c**. Structural alignment of NS1-EDs from interfaces A and B (coloured in red and wheat) and unbound NS1-ED (PDB: 3O9T in cyan) shows that no conformational changes take place upon NS1/TRIM25 complex formation. **d**. W187$_{ED}$ is involved in reciprocal interactions with a symmetry related NS1-ED in the crystal of the TRIM25-CC/NS1-ED complex and is not involved in TRIM25 binding.

a

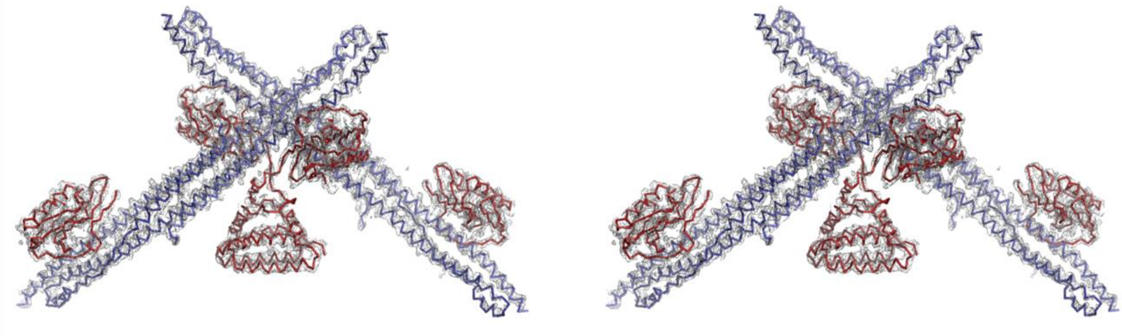

b

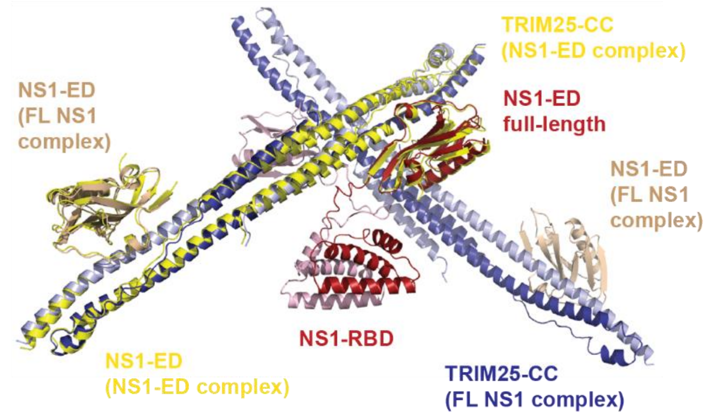

Supplementary Figure 2. The TRIM25-CC/NS1-FL complex.

a. 2Fo-Fc electron density map contoured at 1.2 sigma of the asymmetric unit of the TRIM25-CC/NS1-FL structure in grey mesh with the model represented as ribbon in blue (TRIM25) and red (NS1), shown as a cross-eye stereo image. **b**. Structural alignment of the TRIM25-CC/NS1-ED complex (shown in yellow) with the TRIM25-CC/NS1-FL structure shows no significant structural differences.

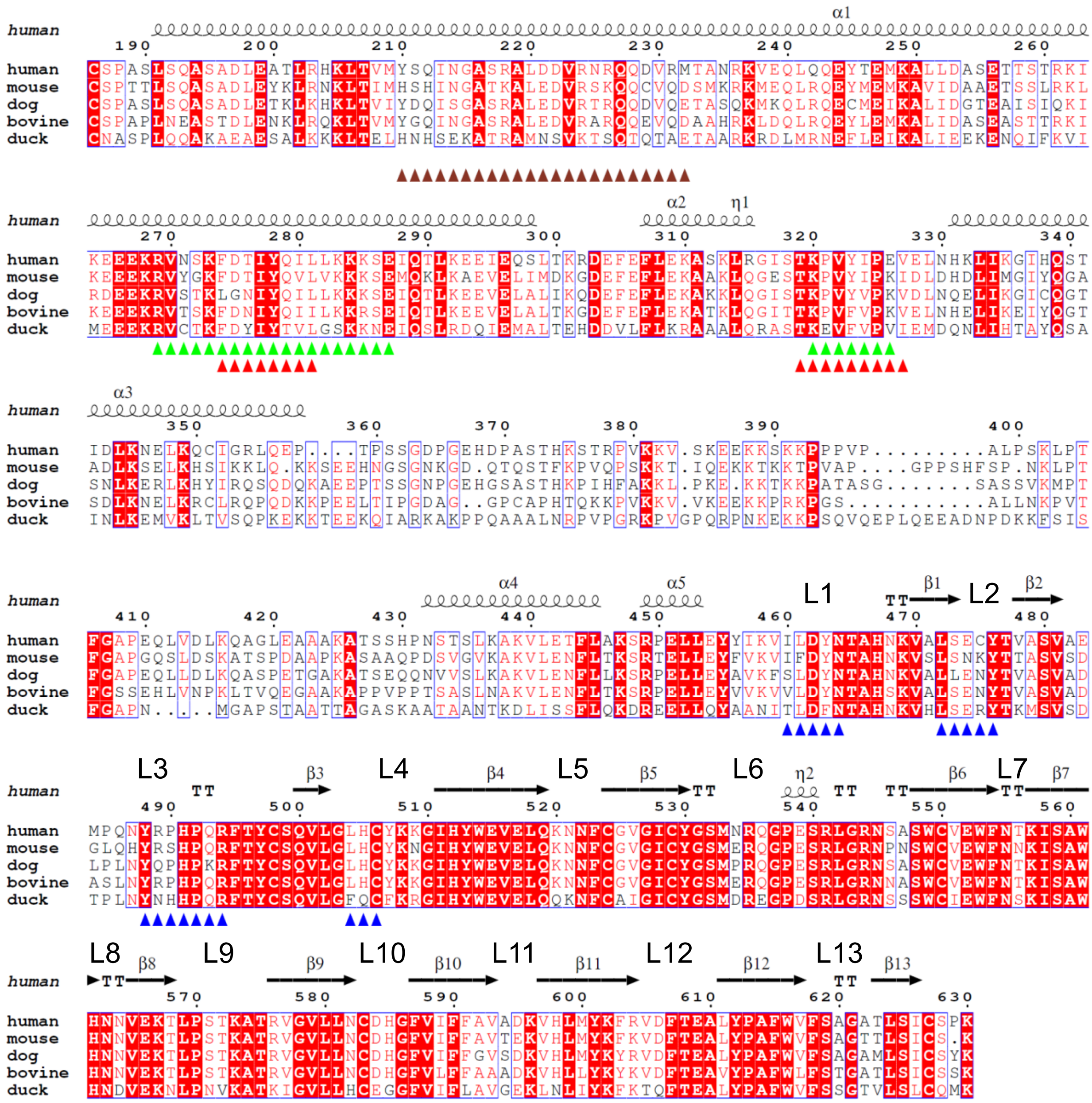

Supplementary Figure 3. Sequence alignment of TRIM25 from different species. Sequence alignment of coiled-coil, linker and PRYSPRY domain of indicated species drawn with ESPRIPT[2]. The secondary structure derive from the CC-PRYSPRY structure determined here is shown above. L1-L13 designate loops in the PRYSPRY domain structure. The footprint of NS1 on CC momomer 1 or 2 is shown by brown or red triangles respectively. The footprint of the PRYSPRY domain on the CC monomer 1 is indicated by green triangles. The footprint of the CC on the PRYSPRY domain is indicated by blue triangles. Footprints were determined by PISA (http://www.ebi.ac.uk/pdbe/pisa/).

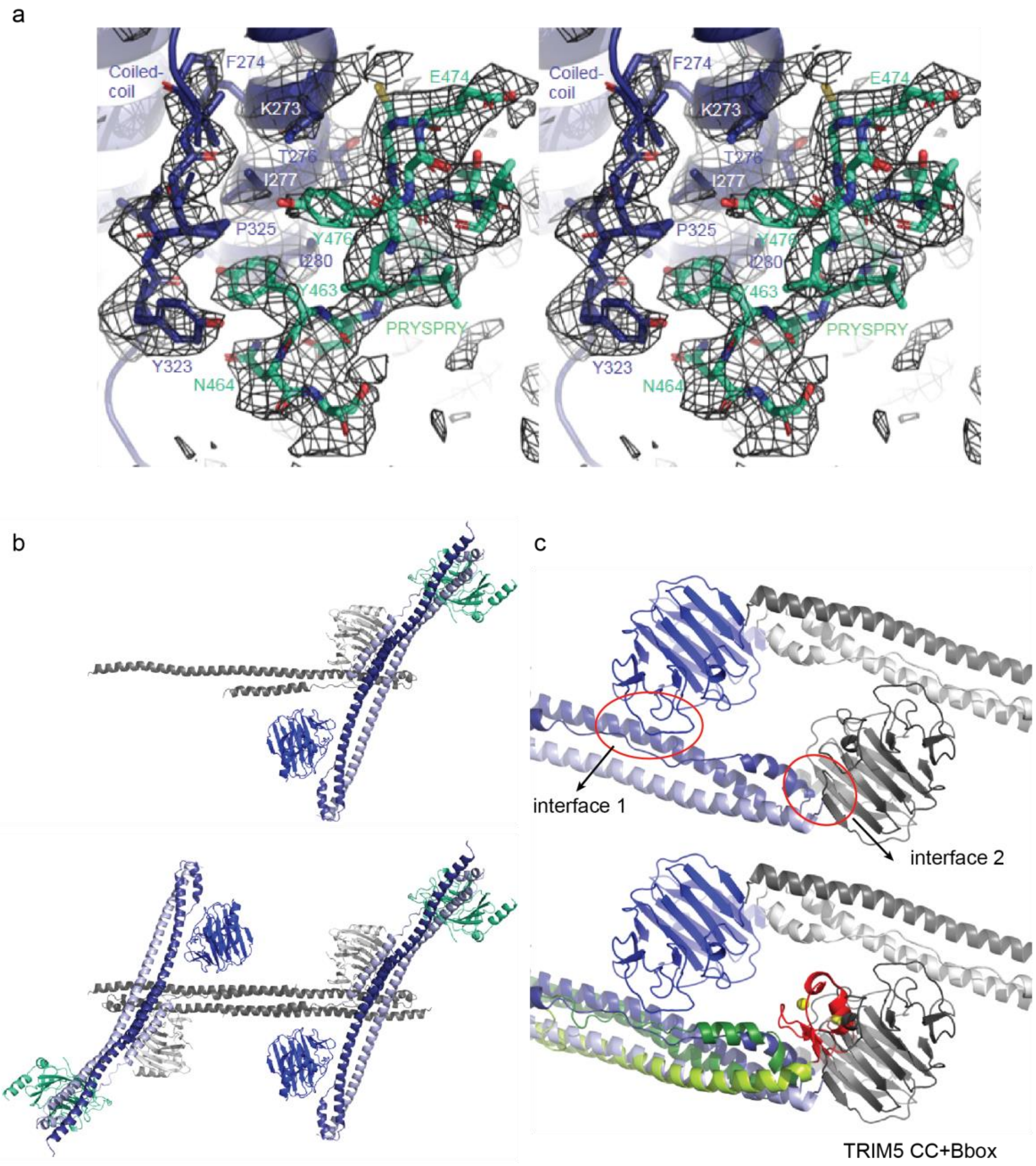

Supplementary Figure 4. Structure of the human TRIM25 CC-PRYSPRY domain.

a. Omit difference map (sharpened with B-factor -15 Å^2) of the interface between the coiled-coil (deep-blue) and the PRYSPY domain (green-cyan) contoured at 2.0 σ. **b**. Asymmetric unit comprising 1.5 dimers (top). The crystallographic 2-fold axis completes the second dimer and crystal symmetry generates a three-dimensional network through the crystal (bottom). **c**. In the crystal, there are two interfaces between the CC and the PRYSPRY domain (top). Interface 1 buries a total surface area of 1430 Å^2 and is described in the main text together with evidence for its biological significance. Interface 2 is with the extremity of the coiled-coil and buries a total surface area of only 749 Å^2. This interface involves Phe305 in the turn at the end of the coiled-coil packing onto a hydrophobic patch on the PRSPRY domain in the

vicinity of Tyr601 and Phe603. However, this is likely an artefact of the crystal packing of the truncated TRIM25 construct lacking the B-boxes, since superposition with the structure of TRIM5 CC (green) with its B-box (red) (PDB:4TN3)[3] suggests that there would be steric clash between the PRYSPRY domain and the B-box (bottom).

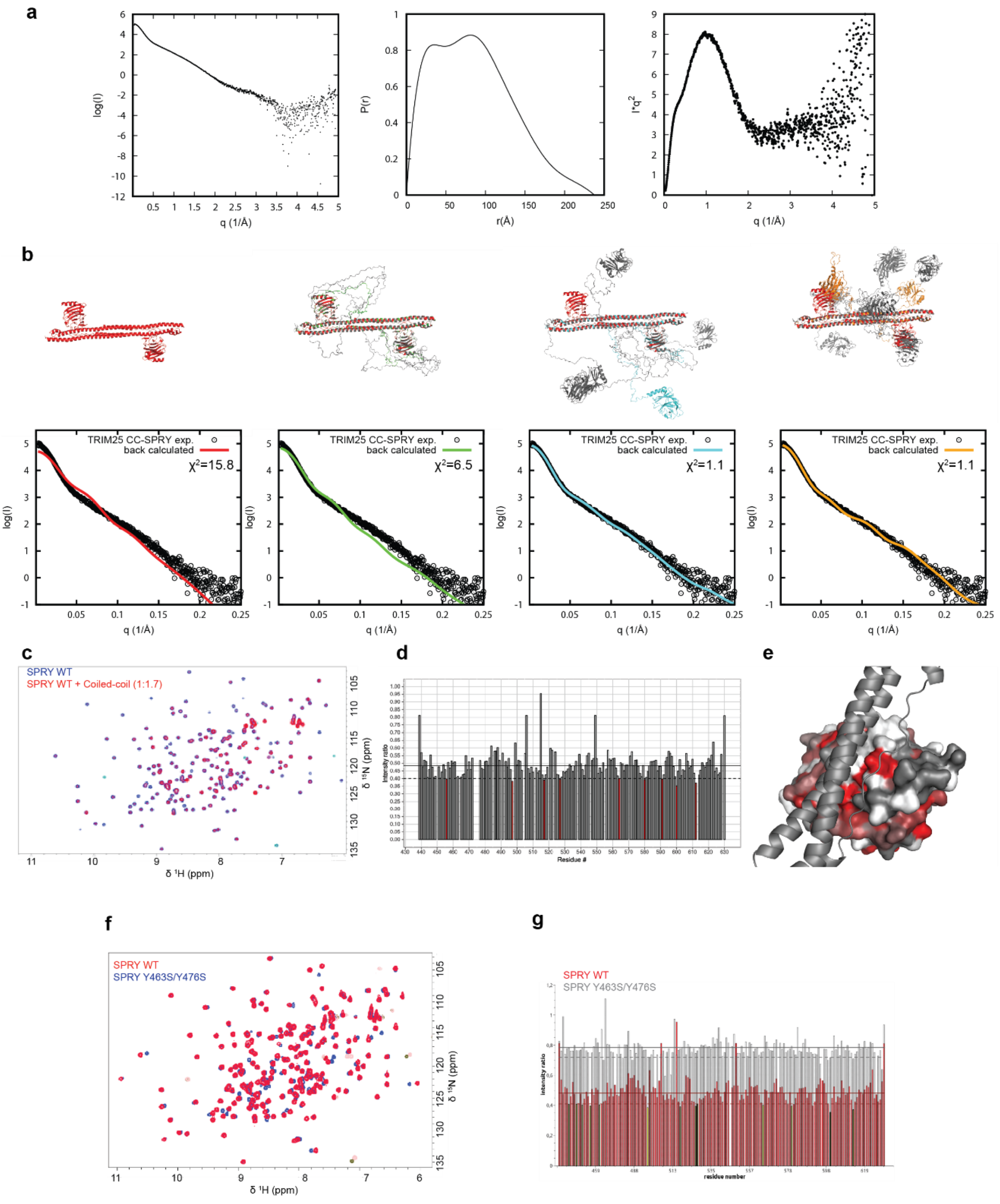

Supplementary Figure 5. The interaction of the coiled-coil and PRYSPRY domains in solution.

a. To validate the crystal structure of the hTRIM25 CC-PRYSPRY domain in solution we performed small-angle X-ray scattering (left: buffer subtracted scattering curve). The pairwise distribution function (middle panel) already indicates that the CC-PRYSPRY domains do not tumble together in solution as the pronounced peak at 35 Å corresponds to an extended PRYSPRY domain and the peak at 100 Å to the CC domain. A D_{max} of > 220 Å also indicates an extended PRYSPRY domain. The Kratky representation (right panel) exhibits a curvature

indicative of a mixture of folded/rigid (sharp maximum at q ≈ 1) and flexible parts (values > 2 $Å^{-1}$ do not decrease to the baseline) corresponding to the linker. **b**. The obtained experimental scattering curve was first fitted against the scattering curve back-calculated from the crystal structure (red line). As expected due to the flexible linker region not resolved in the crystal structure, the calculated scattering curve did not fit the data well with a χ^2 of 15.8 (left). Modelling the disordered conformation of the flexible linker to fit the experimental data against an ensemble of possible structures reduced the χ^2 to 6.5 (green-line, middle-left). The fits were further significantly improved by allowing for dissociation of one or both PRYSPRY domains during structure calculation, resulting in a χ^2 of 1.1 (middle-right, right, cyan and orange line). Selected structures from each ensemble are shown on top of each diagram with similar colour coding and superimposed to the crystal structure (red). From this data, it can be concluded that in solution, in the absence of other binding partners, the PRYSPRY domain is at best weakly bound to the coiled-coil domain. Similar results were found by D'Cruz et al.[4]. **c-e**. NMR titrations between the CC and PRYSPRY domains to test whether there is an interaction in solution at all. ^{15}N-labelled PRYSPRY domain was titrated with the unlabelled coiled-coil (residues 189-379) domain and ^{1}H-^{15}N-HSQCs were recorded to monitor signal intensity changes. Chemical shift perturbations are difficult to observe due to the size of the complex, where peaks corresponding to the bound state would disappear below the noise level. Therefore, to assess binding, ratios of peak intensities between the free and bound state for all assigned residues (175 out of 184 HN-N resonances, 95 %) in the PRYSPRY domain were plotted against the sequence (**d**). After correction for dilution effects a pronounced global decrease by more than two-fold in peak intensity was observed. This is due to line-broadening caused by slower overall tumbling and faster relaxation and is a clear indication of binding. For several residues this effect is stronger, suggestive of direct involvement or being in close proximity to the binding interface (highlighted in red). Some of the residues forming the interface are part of the unassigned region and comparison with the structure indicates that the residues close to the interface are among the most affected, showing that the interaction via the interface observed in the crystal structure is also present in solution (**e**). **f, g**. Mutation of PRYSPRY interface residues Y463 and Y476. To further demonstrate that the binding interface observed in the crystal structure is relevant, two residues located in the interface were mutated to serines (Y463S/Y476S) and the NMR titrations were repeated with exactly the same conditions. That the overall fold of the mutated PRYSPRY domain is not perturbed is shown by comparing ^{1}H-^{15}N-HSQC experiments of wild type and mutant PRYSPRY (**g)**. Despite few chemical shift perturbations due to mutations, peaks are at similar positions and well-dispersed, which proves that the mutant is folded. The interaction with the CC domain is significantly weakened as NMR resonances decrease in signal intensity upon titration not as pronounced as for the wild type (1.25-fold instead of 2-fold).

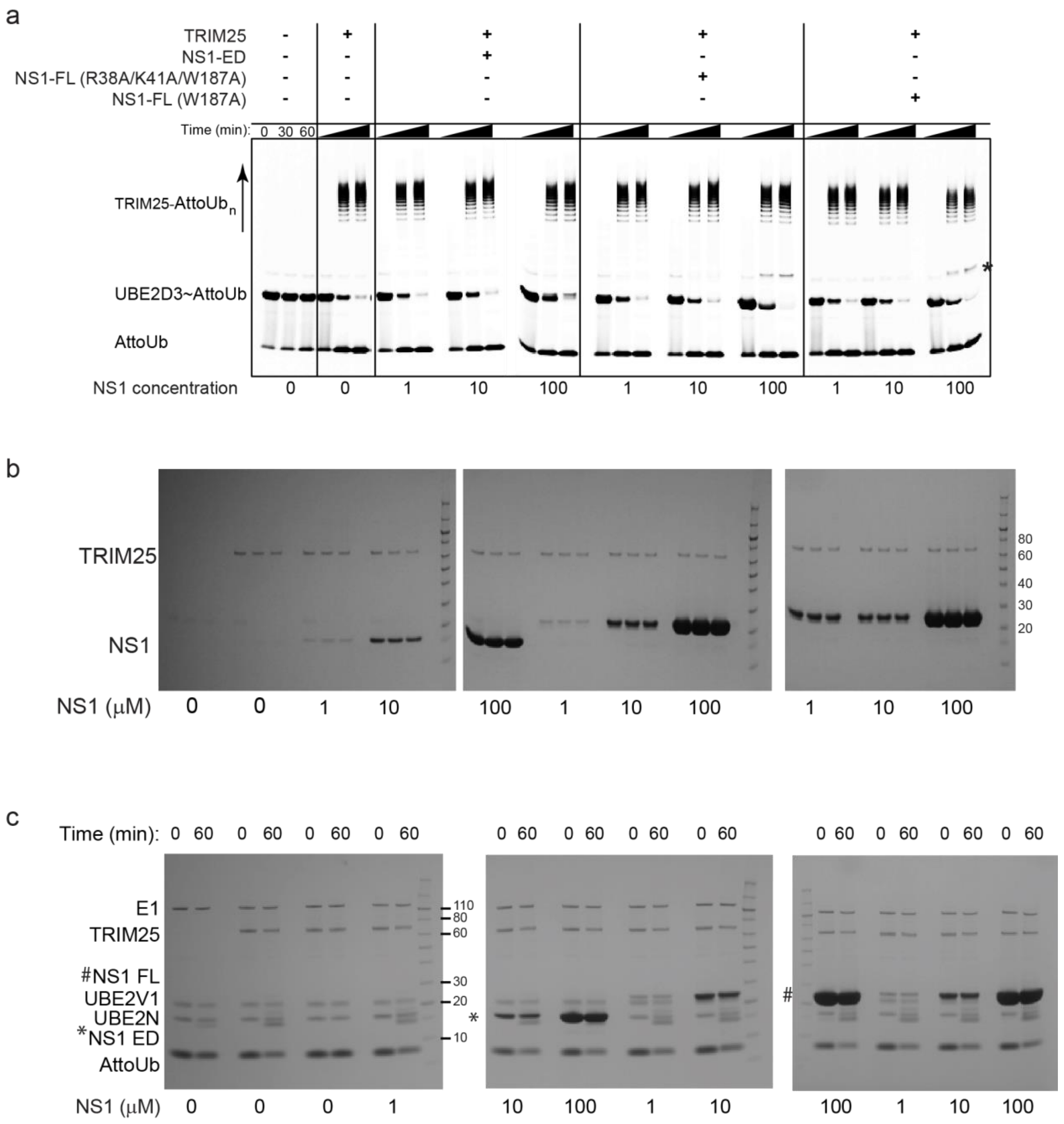

Supplementary Figure 6. NS1 does not affect the intrinsic catalytic ability of TRIM25.

a. UBE2D3~AttoUb discharge assays with TRIM25-FL in the absence or presence of increasing amounts of NS1. Assays were carried out with NS1-ED or NS1-FL (R38A/K41A/W187A or W187A) and the reaction was monitored over 15 min. Gels were scanned with a Typhoon FLA 9500 and the fluorescence converted to black and white. TRIM25 undergoes autoubiquitination under these conditions and FL NS1 becomes mono-ubiquitinated (indicated by asterisk). **b.** InstantBlue stained gels of (a.) including molecular weight markers. **c.** InstantBlue stained gels of Figure 5b including molecular weight markers.

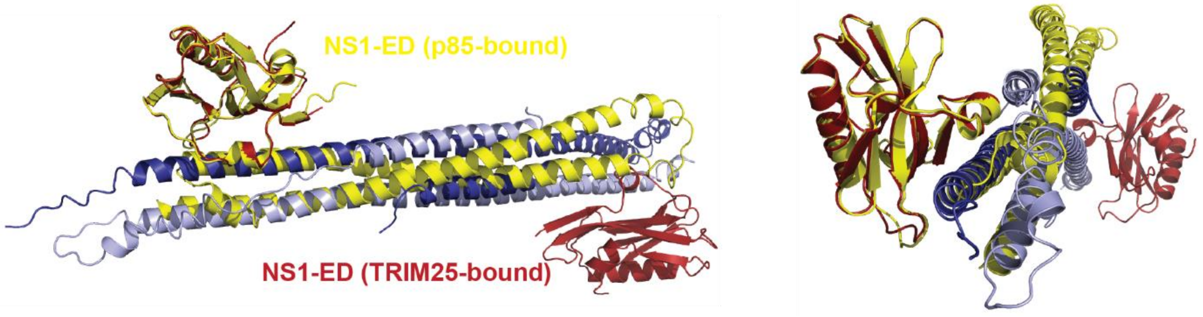

Supplementary Figure 7. Overlap of the NS1-ED complexes of TRIM25-CC and p85β. Overlap of complexes of the effector domain of NS1 bound to TRIM25-CC (ED in red, CC in blue) and p85β (yellow) (PDB: 3L4Q) in two different orientations. The short NS1 α-helix comprising residues 95-99 packs against the target CC region in both complexes. Furthermore, Y89 of NS1 forms hydrogen bonds with TRIM25 as well as p85 β.

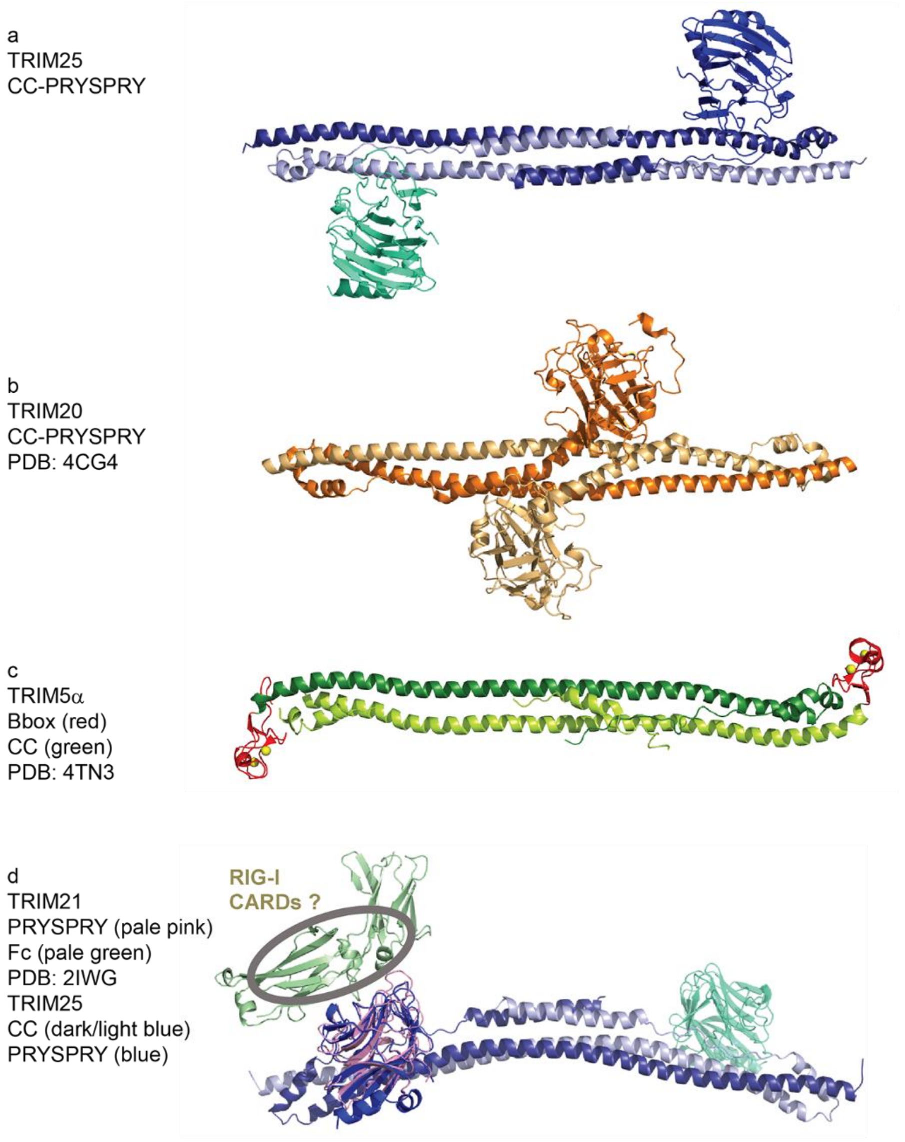

Supplementary Figure 8. Comparison of TRIM25 CC-PRYSPRY with other relevant structures.

a. Human TRIM25 CC-PRYSPRY structure looking down the 2-fold axis of the dimer (this work). The 73 residue linker between the two domains is not visible. **b**. TRIM20 CC-PRYSPRY structure (PDB:4CG4). The shorter structured, but flexible linker between the two domains is visible. **c**. TRIM5α Bbox-CC structure (PDB: 4TN3). In comparison, TRIM25 has a double Bbox, for which there is no structure yet. **d**. Superposition of the TRIM21 PRYSPRY domain-Fc complex (PDB: 2IWG) on the TRIM25 PRYSPRY domain in the CC-PRYSPRY structure. This shows that the equivalent surface that binds the substrate Fc in the case of TRIM21 is available for possible interaction with the RIG-I CARDs (oval) in the case of TRIM25.

Uncropped western blot images related to Figure 4c

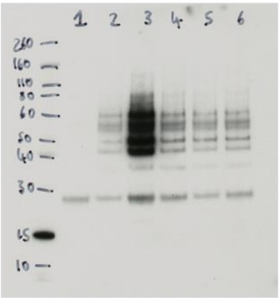

IB: α ubiquitin

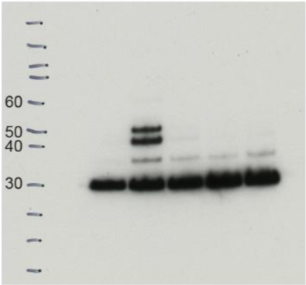

IB: α FLAG

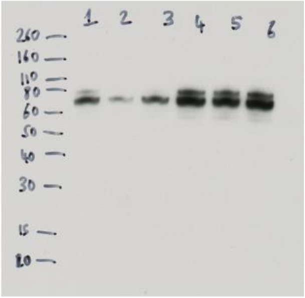

IB: α TRIM25

Uncropped western blot images related to Figure 5a

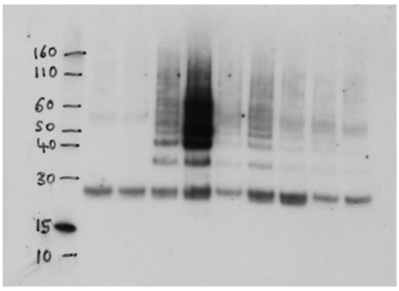

IB: α ubiquitin

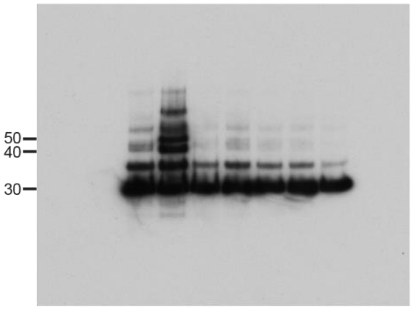

IB: α FLAG

Uncropped western blot image related to Figure 5c

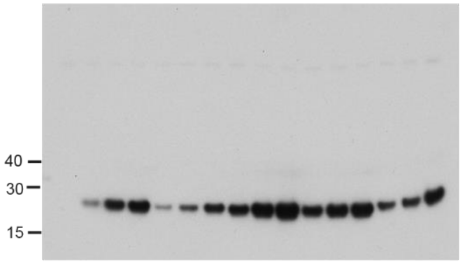

IB: α NS1

Supplementary Figure 9. Uncropped western blot images relating to Figures 4 and 5.

Supplementary References

1 Trewhella, J. *et al.* 2017 publication guidelines for structural modelling of small-angle scattering data from biomolecules in solution: an update. *Acta crystallographica. Section D, Structural biology* **73**, 710-728, doi:10.1107/S2059798317011597 (2017).

2 Gouet, P., Courcelle, E., Stuart, D. I. & Metoz, F. ESPript: analysis of multiple sequence alignments in PostScript. *Bioinformatics* **15**, 305-308 (1999).

3 Wagner, J. M. *et al.* Mechanism of B-box 2 domain-mediated higher-order assembly of the retroviral restriction factor TRIM5alpha. *eLife* **5**, doi:10.7554/eLife.16309 (2016).

4 D'Cruz, A. A. *et al.* Identification of a second binding site on the TRIM25 B30.2 domain. *The Biochemical journal* **475**, 429-440, doi:10.1042/BCJ20170427 (2018).
